# Shifting the boundaries for early caffeine initiation in neonatal practice: Results of a prospective, multicenter study on very preterm infants with respiratory distress syndrome

**DOI:** 10.1371/journal.pone.0189152

**Published:** 2017-12-20

**Authors:** Maria Katarzyna Borszewska-Kornacka, Roman Hożejowski, Magdalena Rutkowska, Ryszard Lauterbach

**Affiliations:** 1 Neonatal and Intensive Care Department, Medical University of Warsaw, Warsaw, Poland; 2 Medical Department, Chiesi Poland Sp. z o.o., Warsaw, Poland; 3 Clinic of Neonatology and Neonatal Intensive Care, Institute of Mother and Child, Warsaw, Poland; 4 Department of Neonatology, Jagiellonian University Medical College, Cracow, Poland; Hopital Robert Debre, FRANCE

## Abstract

**Background:**

There is growing evidence that supports the benefits of early use of caffeine in preterm neonates with RDS; however, no formal recommendations specifying the exact timing of therapy initiation have been provided.

**Objectives:**

We compared neonatal outcomes in infants receiving early (initial dose on the 1st day of life) and late (initial dose on day 2+ of life) caffeine therapy.

**Methods:**

Using data from a prospective, cohort study, we identified 986 infants ≤32 weeks’ gestation with RDS and assessed the timing of caffeine therapy initiation, need for ventilatory support, mortality and incidence of typical complications of prematurity. To adjust for baseline severity, the early and late caffeine groups were propensity score (PS) matched to 286 infants (1:1). Clinical outcomes were compared between the PS-matched groups.

**Results:**

Early treatment with caffeine citrate was associated with a significantly reduced need for invasive ventilation (71.3% vs 83.2%; P = 0.0165) and total duration of mechanical ventilation (mean 5 ± 11.1 days vs 10.8 ± 14.6 days; P = 0.0000) and significantly lower odds of intraventricular hemorrhage (IVH) (OR 0.4827; 95% CI 0.2999–0.7787) and patent ductus arteriosus (PDA) (OR 0.5686; 95% CI 0.3395–0.9523). The incidence of bronchopulmonary dysplasia (BPD) (36.4% vs 45.8%) and rates of moderate and severe BPD were not significantly different between the two groups. The mortality rates were comparable between the two groups (8.6% vs 8.5%, P = ns)

**Conclusion:**

Early caffeine initiation was associated with a decreased need for invasive ventilatory support and lower incidence of IVH and PDA.

## Introduction

Caffeine citrate is a medication commonly used in neonatology and considered the gold standard in the prevention and treatment of apnea of prematurity. This medication is usually used during the first days or sometimes even hours of life up to 34–35 weeks of postconceptional age. No detailed recommendations exist regarding the optimal timing for caffeine therapy initiation.

A meta-analysis published in 2015 reviewed data from 5 publications on early caffeine administration [1]. Whilst all the reviewed studies reported the benefits of earlier vs later initiation of caffeine treatment, they differed in their definitions of “early” treatment [1,2,3,4,5]. Most often “early” treatment was defined as treatment initiated within the first 3 days of life.

However, in clinical practice, the threshold for “early” caffeine treatment is constantly shifting towards earlier initiation. Despite the lack of formal recommendations, in neonatal departments in Poland, caffeine treatment is usually initiated on the first day of life. There are also supporters of initiating caffeine therapy directly after birth while still in the delivery room.

The wide variety of practices in this regard justifies the need for an analysis evaluating whether initiation of treatment during the first 24 hours after birth is more beneficial than initiating during the following days of life. Early therapy is likely to have a positive impact on reducing the incidence of bronchopulmonary dysplasia (BPD) and persistent ductus arteriosus (PDA), shortening the duration of mechanical ventilation and lowering the proportion of children requiring home oxygen therapy [2,5].

## Objective

The objective of our study was to compare the effects of early vs late caffeine citrate administration on clinical outcomes in premature infants ≤ 32 weeks of gestational age with respiratory distress syndrome (RDS).

## Methods

This analysis was based on the data collected in a large, multicenter, prospective study, the NeoPro study, aimed at evaluating adherence to the European Guidelines on the Management of RDS in neonatal departments in Poland. Here, we report the results of an assessment of the timing of caffeine citrate treatment and its impact on clinical outcomes.

Data were collected between November 2014 and December 2015 in 42 level-III and 5 level-II neonatal intensive care units using dedicated questionnaires.

Inclusion criteria were as follows: (1) gestational age ≤ 32 weeks, (2) diagnosis of RDS regardless of the severity of radiological findings on chest X-ray, and (3) need for surfactant treatment. The presence of clinically significant congenital defects was an exclusion criterion.

The NeoPro study was a non-interventional, observational study. Hence, a particular therapeutic strategy was not decided in advance by the study protocol, but reflected standard care in the given center and all the medicinal products were prescribed in the usual manner. Consequently, timing of initiation of caffeine treatment was not protocol-defined but depended upon the routine practice at study sites. On admission to the NICU a written consent with regard to the use of therapeutic procedures and medicinal products was signed by parents or legal guardians. Clinical data was collected anonymously, without any identifying information about the individual patients. The study protocol was approved by the Bioethics Committee of the Medical University of Warsaw (decision AKBE 106/14), in accordance with the principal investigator’s affiliation.

The “early” caffeine treatment group included infants in whom caffeine citrate therapy was initiated during the first 24 hours after birth, and the “late” treatment group included infants in whom therapy was started on the second day of life or after.

Study endpoints included the duration of respiratory support and the incidence of BPD and PDA. As secondary endpoints, we analyzed mortality before hospital discharge and the rates of intraventricular hemorrhage (IVH) and periventricular leukomalacia (PVL).

BPD was diagnosed at the postconceptional age of 36 weeks or at hospital discharge depending on which occurred earlier. Diagnostic criteria included the need for supplemental oxygen for at least 28 days and oxygen dependency at the time of diagnosis (breathing ambient air, mild BPD; <30% oxygen, moderate BPD; and >30% oxygen: severe BPD) [6]. Intra- and periventricular hemorrhages were diagnosed using trans-fontanel ultrasonography performed according to the approved standards and classified using the Papile grading system [7]. The diagnosis of PDA was made based on echocardiography results and treated with ibuprofen when hemodynamically significant. Failure to respond to pharmacological therapy was an indication for surgical ligation [8].

Given that the study was not randomized, we used propensity score matching (PSM) to adjust for the impact of baseline characteristic differences between the “early” and “late” caffeine groups on the final treatment outcomes. The PSM method mimics the randomization process by matching participants in different groups based on baseline covariates. This statistical technique allows for the selection of a control group that is as similar as possible to the study group, with the only difference being "early" or "late" administration of caffeine. The two groups were matched with respect to the following variables: age, gestational age, birth weight, place of birth (inborn/outborn), mode of delivery, 5-minute APGAR score, use of antenatal steroids and need for intubation in the delivery room. For PSM we employed the nearest neighbor without replacement method [9]. To minimize the number of poor matches, a caliper equal to 0.05 of the standard deviation of the propensity score was used.

For comparisons between independent variables, the two-sided Student’s t-test (in case of normal distribution) or U-Mann-Whitney test (non-normal distribution) was used. To compare neonatal outcomes (incidence of typical complications of prematurity), we used Yate’s continuity corrected chi-square test or Fisher’s exact test. Confidence intervals (95%) for the odds ratios were calculated using Wald's method. The level of significance for all comparisons was established at 5%.

The analysis was performed with R statistical software (version 3.2), Foundation for Statistical Computing, Vienna, Austria. R MatchIt package (version 2.4) was used for propensity score analysis.

## Results

Of the 986 infants who met inclusion criteria, 888 (90%) received treatment with caffeine citrate. A complete dataset was obtained for 844 newborns. The group included 54.1% males, and most infants were inborn (89,3%). The median gestational age was 28 weeks (IQR, 27–30 days), and the median body weight was 1095 g (IQR, 840–1370 g). Extremely low body weight (ELBW) infants (< 1000 g) constituted 41,6% of the group.

**Fig 1:**
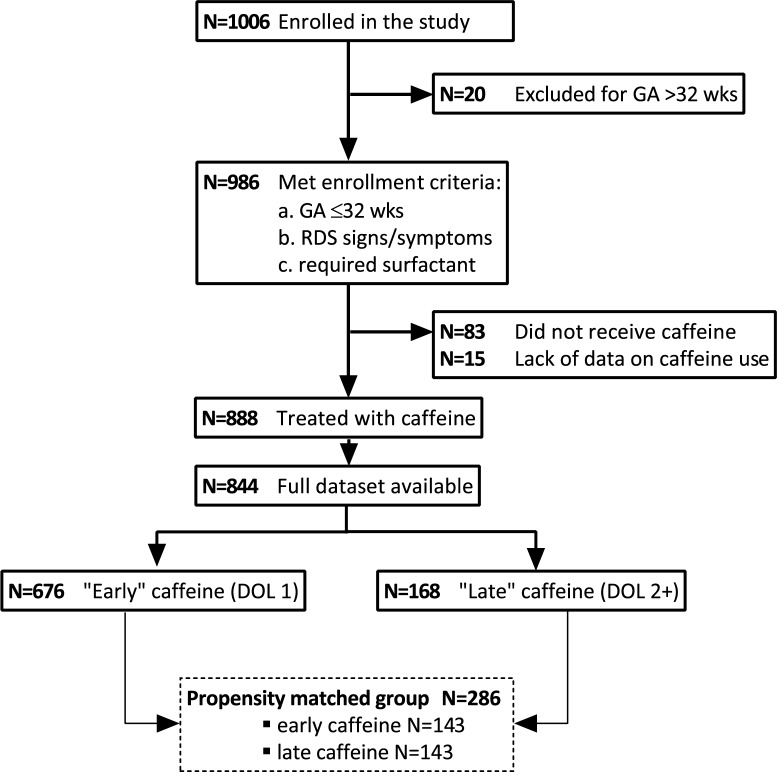
Patient flow diagram. DOL = Day of Life.

Of the 844 infants, 676 (80,1%) received caffeine treatment during the first 24 hours of life (“early” caffeine) and 168 (19,9%) received caffeine treatment on the second day of life of later (“late” caffeine); [Fig 1].

In the latter group, the median (IQR) time to treatment initiation was 4 days (2–10 days).

The two groups differed significantly in antenatal steroid exposure (82.3% vs 71.5% in the “early” and “late” group, respectively; P = 0.0026) and gestational age, which was lower in the “early” caffeine group (median 28.1 vs 29.0 weeks; P = 0.0680).

After employing the PSM procedure, two equally numbered groups of 143 infants were formed that had similar baseline characteristics and no significant differences in any of the matched variables (Table 1). Clinical outcomes were compared between the propensity matched cohorts.

**Table 1:** Clinical characteristics of the study cohort (N = 844) overall and within groups paired using the PSM method (N = 286).

Characteristics	All infants			Propensity-matched infants		
	Early caffeine	Late caffeine	P value	Early caffeine	Late caffeine	P value
	N = 676	N = 168		N = 143	N = 143	
**GA, weeks**						
Mean ±SD	28.2 ±2.2	28.5 ±2.4	0.0680	28.6 ±2.1	28.5 ±2.4	0.9618
Median (IQR)	28 (27–30)	29 (27–30)		29 (27–30)	29 (27–30)	
**Birth weight, g**						
Mean ±SD	1118 ±357	1155 ±404	0.4397	1174 ±357	1168 ±406	0.8190
Median (IQR)	1090 (840–1345)	1100 (845–1455)		1130 (895–1450)	1100 (850–1485)	
**Sex**						
Male, n (%)	364 (54.3)	88 (53.0)	0.8277	77 (53.8)	76 (53.8)	1.0000
**Place of birth**						
Inborn, n(%)	576 (88.9)	147 (90.7)	0.5899	129 (90.2)	133 (93.0)	0.5223
Outborn, n(%)	72 (11.1)	15 (9.3)		14 (9.8)	10 (7.0)	
**Mode of delivery**						
C-section, n(%)	533 (79.6)	134 (80.7)	0.8194	111 (77.6)	115 (80.4)	0.6631
Vaginal birth, n(%)	137 (20.4)	32 (19.3)		32 (22.4)	28 (19.6)	
**Apgar at 5 min.**						
Mean ±SD	6.5 ±1.9	6.5 ±2.3	0.6388	6.6 ±2.1	6.5 ±6.5	0.9543
Median (IQR)	7 (6–8)	7 (6–8)		7 (6–8)	7 (6–8)	
**Antenatal steroid use**						
N (%)^#^	550 (82.3)	118 (71.5)	0.0026	106 (74.1)	106 (74.1)	1.0000
**Intubation in the DR**						
N (%)	349 (52.2)	91 (54.2)	0.7058	76 (53.1)	77 (53.8)	1.0000
**Caffeine therapy, days**						
Mean ±SD	37.8 ±23.3	24.8 ±19.3	0.0000	35.1 ±25	24.3 ±19.7	0.0000
Median (IQR)	35 (20–52)	21.5 (9.8–36)		31 (19–48)	21 (9–35)	

# data on antenatal steroids were unavailable for 11 neonates, including 8 infants in early caffeine and 3 infants in late caffeine groups.

### Respiratory support

In PS-matched infants, “early” caffeine receipt was associated with a decreased need for invasive ventilation (71.3% vs 83.2%; P = 0.0165) and significantly shorter total duration of mechanical ventilation relative to “late” caffeine (mean 5 ±11.1 days vs 10.8 ±14.6 days; P = 0.0000). A similar difference was not observed in the duration of non-invasive respiratory support, which did not differ significantly between groups (mean 15.9 ±15.3 days vs 13.7 ±15.2 days; P = 0.1595).

### BPD

Although seeming to show a trend, the incidence of BPD (36.4% vs 45.8%) and rates of moderate BPD (5,9% vs 12.1%) and severe BPD (3.4% vs 4.7%) were not statistically significant between the ‘early’ and the ‘late’ treatment groups [Table 2].

**Table 2:** Study endpoints in PS-matched infants.

	Early caffeine	Late caffeine	P value
	N = 143	N = 143	
**Length of hospital stay, days**			
Mean ±SD	58.1 ±38.7	53.8 ±29.7	0.2413
Median (IQR)	54 (40–72)	49 (32–73)	
**Need for MV**			
N (%)	102 (71.3)	119 (83.2)	**0.0165**
**Mechanical ventilation, days**			
Mean ±SD	5 ±11.1	10.8 ±14.6	**0.0000**
Median (IQR)	1.0 (0.0–4.0)	4.3 (1.0–15.9)	
**Non-invasive ventilation, days**			
Mean ±SD	15.9 ±15.3	13.7 ±15.2	0.1595
Median (IQR)	10 (3–25)	7 (2–23)	
**Death**			
N (%)	12 (8.6)	12 (8.5)	1.0000
**BPD**			
No BPD, N (%)	75 (63.6)	58 (54.2)	0.3117
Mild BPD, N (%)	32 (27.1)	31 (29.0)	
Moderate BPD, N (%)	7 (5.9)	13 (12.1)	
Severe BPD, N (%)	4 (3.4)	5 (4.7)	
**IVH**			
N (%)	59 (42.1)	83 (60.1)	**0.0039**
Grade I, N (%)	14 (26.9)	25 (30.9)	0.7036
Grade II, N (%)	26 (50.0)	36 (44.4)	
Grade III, N (%)	5 (9.6)	12 (14.8)	
Grade IV, N (%)	7 (13.5)	8 (9.9)	
**PDA**			
N (%)	35 (25)	51 (37)	**0,0427**
**Pharmacological closure of PDA**			
N (%)	14 (43.7)	18 (37.5)	0.7444
**Surgical ligation of PDA**			
N (%)	4 (12.5)	8 (16.7)	0.8479
**PVL**			
N (%)	4 (2.9)	11(8)	0.0610

**Fig 2:**
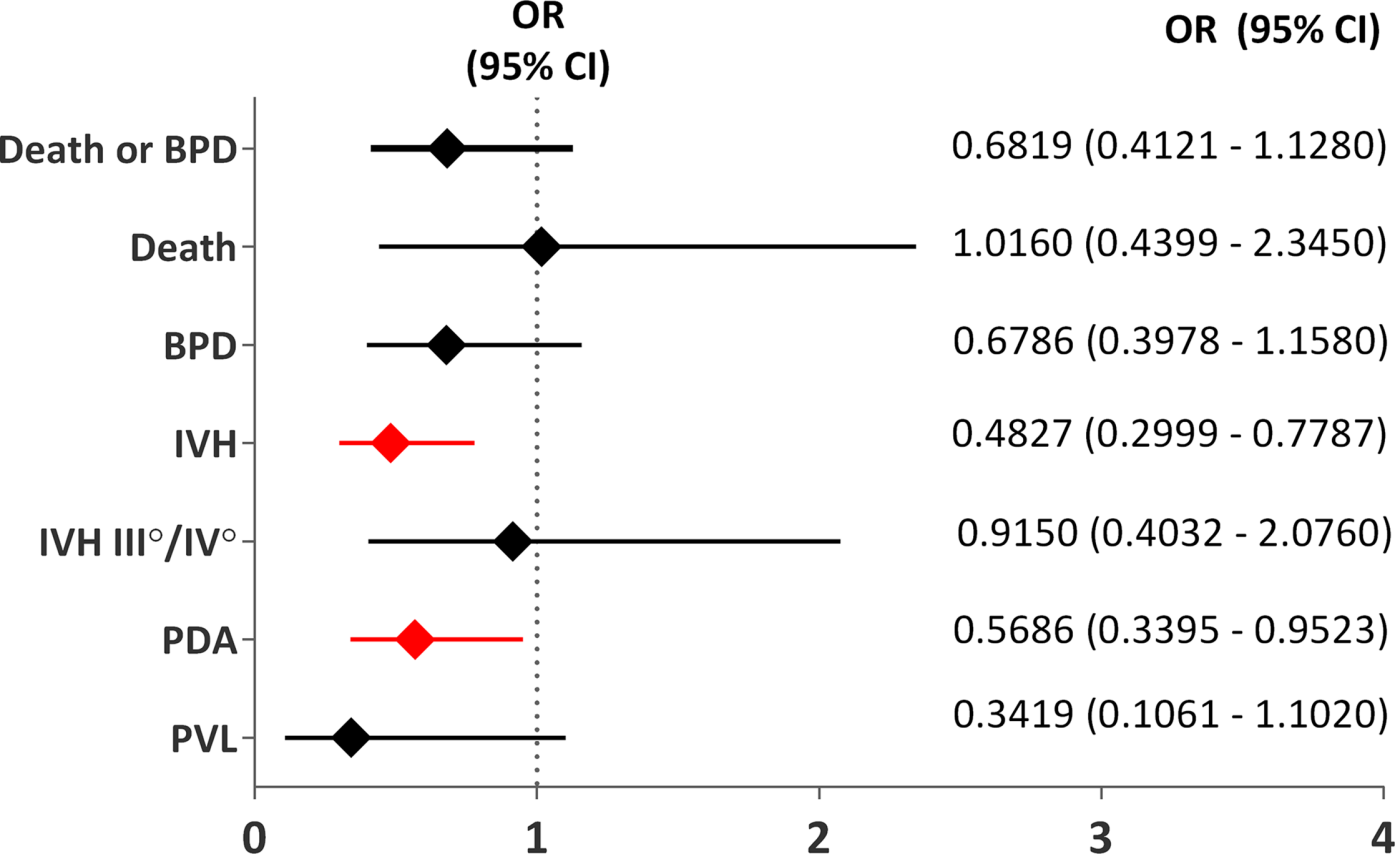
Comparison of the effects of “early” and “late” caffeine therapy on the risk of typical complications of prematurity.

“Early” caffeine was associated with a significantly lower incidence of PDA (25% vs 37%; P = 0.0427) and significantly reduced odds of PDA (OR 0.5686; 95% CI 0.3395–0.9523) relative to “late” caffeine. Nevertheless, no statistically significant differences were observed in the rates of pharmacotherapy for or surgical ligation of PDA (Fig 2).

### Secondary endpoints

IVHs were observed more often (60.1%) in the late therapy group than in the early therapy group (42.1%; P = 0.0039). Early treatment initiation was associated with a significantly reduced risk of IVHs (OR 0.4827; 95% CI 0.2999–0.7787) but had no impact on the occurrence of the most severe forms of the disease (grade 3 and 4) when compared with late initiation. PVL was diagnosed in 2.9% infants in the “early” caffeine group and 8% infants in the “late” caffeine group (P = 0.0610) (Table 2). The rates of mortality before hospital discharge were almost identical between groups (8.6% vs 8.5%; P = ns)

## Discussion

### Main finding

This paper is the first large study to investigate the effect of very early caffeine administration on the outcomes of infants less than 32 weeks gestation. We have shown that administering caffeine in the first 24 hours of life significantly decreased the need for, and duration of, invasive ventilation compared to matched infants starting caffeine treatment on day two or later. “Early” treatment also significantly reduced the incidence of both intraventricular hemorrhage and persistent ductus arteriosus.

### Previous work

Although caffeine citrate has been used in the prevention and treatment of apnea of prematurity for more than 30 years, its beneficial effects on general morbidity and future psychomotor development have only recently been documented [2,10].

The 2013 European recommendations emphasized the role of the timing of caffeine initiation, suggesting that earlier treatment initiation is associated with more beneficial effects [11]. However, there have been no specific recommendations made regarding timing of initiation or duration of treatment.

Some benefits of very early caffeine administration were documented in a randomized, double-blind trial conducted by Katheria et al. in 2015; however, this pilot study only evaluated a very small group of 21 infants [12]. Eleven of those newborns received caffeine citrate during the first 2 hours of life, while the remainder started treatment later (at 12 hours). Lower numbers of infants who received very early treatment required intubation in the first 12 hours of life (3 vs 7 infants) or needed treatment for PDA (2 vs 4 infants). The authors found no difference in the frequency of intraventricular hemorrhage and, surprisingly, in this study infants in the ‘early’ group required more days of mechanical ventilation. The timeframes used in this study differ from ours–making the results not directly comparable. However, similar to this study, we found fewer infants required mechanical ventilation (71.3% vs 83.2%; P = 0.0165) or developed a PDA; however, we also found that earlier treatment halved the number of days on invasive ventilation, and lowered rates of IVH.

A publication by the Canadian Neonatal Network in 2015 reported retrospective data on 5101 newborns from 29 sites [13]. The authors compared outcomes for infants receiving caffeine either within the first two days of life or after the third day. Despite the longer timeframes, in agreement with our results, they found reduced numbers of PDAs (OR, 0.74; 95% CI 0.62 to 0.89) in the early treatment group. This large study also reported a significant reduction in BPD (OR 0.81; 95% CI 0.67 to 0.98).

Similar findings were reported in 2014 by both Dobson et al. [14] and a group from Philadelphia under the leadership of Taha [15]. Both groups looked at caffeine administration at 0–2 days versus day 3 or later. Both groups found infants receiving caffeine at day three or later were significantly more likely to develop BPD then those receiving it earlier (Dobson et al. OR, 0.68; 95% CI, 0.63–0.73; Taha OR 0.69; 95% CI 0.58 to 0.82; P <0.001). Although our study showed a trend towards reduced BPD, this was not significant.

Few studies have looked at the effect of timing of caffeine administration on IVH. In their small randomized study, Katheria et al. [12] were unable to demonstrate a significant difference. Consistent with our results, Taha et al. reported a lower incidence of severe IVH in early relative to late treatment groups [15]. Disappointingly, in our study we found that although IVH was decreased overall (OR 0.48; 95% CI 0.29 to 0.78), hemorrhages of the greatest severity (grade III or IV) occurred with comparable frequency (23.1% vs 24.7%) in both groups. We also observed a trend towards reduced numbers of patients with PVL (2.9% vs 8%; P = 0.0610) in the ‘early’ versus the ‘late’ treatment group. This finding is in broad agreement with the results of the meta-analysis by Park et al. [1] which found a reduction in PVL in infants treated with caffeine within the first two days of life (P<0.001; OR 0,560; CI 0.494 to 0.635). We would expect that, similar to the findings of Schmidt et al. [16,17], this will translate into a reduced incidence of cerebral palsy, although the length of our study means we cannot answer this question.

### Possible mechanism of action

Caffeine is known to stimulate regular breathing, and increase sensitivity to carbon dioxide [18]. This is likely to improve the success of non-invasive ventilation and lead to the observed decreased need for, and reduced duration of, invasive ventilation. This, along with the anti-inflammatory properties of caffeine [19] may contribute to the improved respiratory outcomes in infants receiving treatment.

The mechanisms behind the ‘non-respiratory’ effects of caffeine treatment continue to be debated. Lower rates of intraventricular hemorrhage may result from improvements in cerebral blood flow, in turn due to fewer periods of intermittent hypoxia which are observed with caffeine treatment [20].

Although observed in several trials, the decreased incidence of PDA is more difficult to explain. Mechanisms including improved hemodynamics [21] and the prostaglandin antagonistic properties of caffeine [22] are possibilities. Further research is needed to clearly understand the relationship of PDA to caffeine administration.

### Limitations of this study

Although prospectively enrolled, this was an observational study, not a randomized trial. Treatment therefore reflects local practice, and outcomes may have been influenced by confounding factors. Given the study involved infants treated at multiple locations, this is of course a significant concern, in particular treatment may have differed between level 2 and level three centers. We believe our findings remain valid for two reasons. Firstly, the use of propensity scores imitates randomization as closely as possible, meaning baseline characteristics of infants are similar. Secondly, the large size of the cohort minimizes the impact of confounding influences from multiple treatment centers.

This study is also limited by duration; by focusing on the early effects of caffeine treatment we are unable to comment on any longer-term effects.

### Strengths of this study

This study represents a significant group of infants, and is the largest report on the outcomes of caffeine treatment given within the first day of life. Prospective enrollment and propensity matching strengthen the certainty of our findings.

### Implications for practice

This study adds to the previous body of work showing the benefits of early administration of caffeine to preterm infants. In agreement with previous work we have shown that early caffeine reduces the incidence of persistent ductus arteriosus and intraventricular hemorrhage, as well as reducing the need for invasive ventilation.

Importantly, this paper has narrowed down the timeframe for ‘early’ administration, demonstrating additional benefits for treatment started during the first 24 hours of life.

In light of these results, we recommend starting caffeine treatment in the first 24 hours for infants less than 32 weeks gestation who required surfactant treatment.

## Conclusions

1. Initiating caffeine citrate therapy within the first 24 hours of life in newborns aged ≤32 gestational weeks who required surfactant treatment was associated with an average duration of mechanical ventilation that was 5 days less than that observed in infants with later therapy initiation.
2. Early treatment with caffeine citrate was associated with a statistically significant reduction in the total rates of PDA and IVHs; however, lesser reductions were observed in the higher grades of IVH.
3. On the basis of our findings, we recommend that caffeine treatment for suitable infants should be started within the first 24 hours of life.

## Supporting information

S1 Table Neonatal outcomes in propensity-matched cohorts. (XLSX)

## References

[1] Park HW, Lim G, Chung SH, Chung S, Kim KS, Kim SN. Early Caffeine Use in Very Low Birth Weight Infants and Neonatal Outcomes: A Systematic Review and Meta-Analysis. J Korean Med Sci. 2015 12;30(12):1828–35. doi: 10.3346/jkms.2015.30.12.1828 26713059 10.3346/jkms.2015.30.12.1828 PMC4689828

[2] Schmidt B, Roberts RS, Davis P, Doyle LW, Barrington KJ, Ohlsson A, et al Caffeine for Apnea of Prematurity Trial Group. Caffeine therapy for apnea of prematurity. N Engl J Med. 2006 5 18;354(20):2112–21. doi: 10.1056/NEJMoa054065 16707748 10.1056/NEJMoa054065

[3] Morton SU, Smith VC. Treatment options for apnoea of prematurity. Arch Dis Child Fetal Neonatal Ed. 2016 7;101(4):F352–6 doi: 10.1136/archdischild-2015-310228 27010019 10.1136/archdischild-2015-310228

[4] Dobson NR, Patel RM, Smith PB, Kuehn DR, Clark J, Vyas-Read S, et al Trends in caffeine use and association between clinical outcomes and timing of therapy in very low birth weight infants. J Pediatr. 2014 5;164(5):992–998. Erratum in: J Pediatr. 2014 May;164(5):1244. doi: 10.1016/j.jpeds.2013.12.025 24461786 10.1016/j.jpeds.2013.12.025 PMC3992195

[5] Henderson-Smart DJ, De Paoli AG. Prophylactic methylxanthine for prevention of apnoea in preterm infants. Cochrane Database of Systematic Reviews 2010, Issue 12. Art. No.: CD000432. 10.1002/14651858.CD000432.pub2 10.1002/14651858.CD000432.pub2 PMC7032541 21154344

[6] Walsh MC. (2008). Definition and predictors of bronchopulmonary dysplasia In Bancalari E, Polin R (Ed.): *The newborn lung* (pp. 233–240). New York, NY: Elsevier Saunders

[7] Papile LA, Burstein J, Burstein R, Koffler H. Incidence and evolution of subependymal and intraventricular hemorrhage: a study of infants with birth weights less than 1,500 gm. J Pediatr. 1978 4;92(4):529–34. 305471 10.1016/s0022-3476(78)80282-0

[8] Mitra S, Rønnestad A, Holmstrøm H. Management of patent ductus arteriosus in preterm infants—where do we stand? Congenit Heart Dis. 2013 Nov-Dec;8(6):500–12. doi: 10.1111/chd.12143 24127861 10.1111/chd.12143

[9] Ho D.E., Imai K., King G., Stuart E.A. MatchIt: Nonparametric preprocessing for parametric causal inference. J Stat Softw 2011;42:1–18

[10] Picone S, Bedetta M, Paolillo P. Caffeine citrate: when and for how long A literature review. J Mater Fetal Neonatal Med 2012;25:11–14 10.3109/14767058.2012.712305 23016611

[11] Sweet DG, Carnielli V, Greisen G, Hallman M, Ozek E, Plavka R, et al; European Association of Perinatal Medicine. European consensus guidelines on the management of neonatal respiratory distress syndrome in preterm infants—2013 update. Neonatology. 2013;103(4):353–68. doi: 10.1159/000349928 23736015 10.1159/000349928

[12] Katheria AC, Sauberan JB, Akotia D, Rich W, Durham J, Finer NN. A Pilot Randomized Controlled Trial of Early versus Routine Caffeine in Extremely Premature Infants. Am J Perinatol. 2015 7;32(9):879–86 doi: 10.1055/s-0034-1543981 25607226 10.1055/s-0034-1543981

[13] Lodha A, Seshia M, McMillan DD, Barrington K, Yang J, Lee SK, et al; Canadian Neonatal Network. Association of early caffeine administration and neonatal outcomes in very preterm neonates. JAMA Pediatr. 2015 1;169(1):33–8 doi: 10.1001/jamapediatrics.2014.2223 25402629 10.1001/jamapediatrics.2014.2223

[14] Dobson NR, Patel RM, Smith PB, Kuehn DR, Clark J, Vyas-Read S, et al Trends in caffeine use and association between clinical outcomes and timing of therapy in very low birth weight infants. J Pediatr. 2014 5;164(5):992–998.e3. doi: 10.1016/j.jpeds.2013.12.025 24461786 10.1016/j.jpeds.2013.12.025 PMC3992195

[15] Taha D, Kirkby S, Nawab U, Dysart KC, Genen L, Greenspan JS, et al Early caffeine therapy for prevention of bronchopulmonary dysplasia in preterm infants. J Matern Fetal Neonatal Med. 2014 11;27(16):1698–702. doi: 10.3109/14767058.2014.885941 24479608 10.3109/14767058.2014.885941

[16] Schmidt B, Roberts RS, Davis P, Doyle LW, Barrington KJ, Ohlsson A, et al; Caffeine for Apnea of Prematurity Trial Group. Long-term effects of caffeine therapy for apnea of prematurity. N Engl J Med. 2007 11 8;357(19):1893–902. doi: 10.1056/NEJMoa073679 17989382 10.1056/NEJMoa073679

[17] Schmidt B, Anderson PJ, Doyle LW. Survival without disability to age 5 years after neonatal caffeine therapy for apnea of prematurity. JAMA 2012;307:275–282 doi: 10.1001/jama.2011.2024 22253394 10.1001/jama.2011.2024

[18] Aranda JV, Turmen T, Davis J, Trippenbach T, Grondin D, Zinman R, et al Effect of caffeine on control of breathing in infantile apnea. J Pediatr. 1983;103:975–978 6644439 10.1016/s0022-3476(83)80735-5

[19] Tunc T, Aydemir G, Karaoglu A, Cekmez F, Kul M, Aydinoz S, et al Toll-like receptor levels and caffeine responsiveness in rat pups during perinatal period. Regul Pept. 2013;182:41–44. doi: 10.1016/j.regpep.2012.12.016 23313844 10.1016/j.regpep.2012.12.016

[20] Rhein LM, Dobson NR, Darnall RA, Corwin MJ, Heeren TC, Poets CF, et al Caffeine Pilot Study Group. Effects of caffeine on intermittent hypoxia in infants born prematurely: a randomized clinical trial. JAMA Pediatr. 2014 3; 168(3):250–7 doi: 10.1001/jamapediatrics.2013.4371 24445955 10.1001/jamapediatrics.2013.4371

[21] Soloveychik V, Bin-Nun A, Ionchev A, Sriram S, Meadow W. Acute hemodynamic effects of caffeine administration in premature infants. J Perinatol. 2009;29:205–208 doi: 10.1038/jp.2008.193 19052555 10.1038/jp.2008.193

[22] Manku MS, Horrobin DF. Chloroquine, quinine, procaine, quinidine, tricyclic antidepressants, and methylxanthines as prostaglandin agonists and antagonists. Lancet. 1976 11 20;2(7995):1115–7. 62951 10.1016/s0140-6736(76)91090-4

